# The roles of osteocytes in alveolar bone destruction in periodontitis

**DOI:** 10.1186/s12967-020-02664-7

**Published:** 2020-12-11

**Authors:** Xiaofei Huang, Mengru Xie, Yanling Xie, Feng Mei, Xiaofeng Lu, Xiaoshuang Li, Lili Chen

**Affiliations:** 1 Department of Stomatology, Union Hospital, Tongji Medical College, Huazhong University of Science and Technology, Wuhan, 430022 China; 2 Hubei Province Key Laboratory of Oral and Maxillofacial Development and Regeneration, Wuhan, 430022 China

**Keywords:** Osteocyte, Periodontitis, RANKL, Sclerostin, Senescence, Apoptosis

## Abstract

Periodontitis, a bacterium-induced inflammatory disease that is characterized by alveolar bone loss, is highly prevalent worldwide. Elucidating the underlying mechanisms of alveolar bone loss in periodontitis is crucial for understanding its pathogenesis. Classically, bone cells, such as osteoclasts, osteoblasts and bone marrow stromal cells, are thought to dominate the development of bone destruction in periodontitis. Recently, osteocytes, the cells embedded in the mineral matrix, have gained attention. This review demonstrates the key contributing role of osteocytes in periodontitis, especially in alveolar bone loss. Osteocytes not only initiate physiological bone remodeling but also assist in inflammation-related changes in bone remodeling. The latest evidence suggests that osteocytes are involved in regulating bone anabolism and catabolism in the progression of periodontitis. The altered secretion of receptor activator of NF-κB ligand (RANKL), sclerostin and Dickkopf-related protein 1 (DKK1) by osteocytes affects the balance of bone resorption and formation and promotes bone loss. In addition, the accumulation of prematurely senescent and apoptotic osteocytes observed in alveolar bone may exacerbate local destruction. Based on their communication with the bloodstream, it is noteworthy that osteocytes may participate in the interaction between local periodontitis lesions and systemic diseases. Overall, further investigations of osteocytes may provide vital insights that improve our understanding of the pathophysiology of periodontitis.

## Introduction

Periodontitis is a complicated, multifaceted process that results in the disorganization of the underlying ligament and alveolar bone. Studies on the mechanisms of periodontitis have always focused on alveolar bone loss, especially the function of osteoclasts and osteoblasts in this process. However, the past few years have witnessed a substantial increase in our understanding of the capacities of osteocytes, and the notion that they are in a state of inactivity has been rejected. An increasing number of studies have revealed a key role of osteocytes in physiological and pathological skeletal events, including periodontitis. The intention of this review is to summarize the role of osteocytes in the remodeling of alveolar bone in periodontitis on the basis of existing evidence in order to provide new insights for future studies of the mechanisms underlying alveolar bone loss in periodontitis and the clinical treatment of periodontitis.

## Periodontitis and alveolar bone loss

Periodontitis has become the most common and consequential oral disease and is among the most important clinical conditions that have attracted considerable attention in the public health field [1]. From 1990 to 2010, the prevalence of severe periodontitis in the entire population remained stable at 11.2% [2]. Periodontitis is characterized by the progressive breakdown of supportive periodontal tissues, especially the loss of alveolar bone. As lesions develop, the height of the alveolar bone gradually decreases, and the tooth foundation becomes weak, resulting in exposed tooth roots and loosened teeth, which, if left untreated, eventually result in tooth loss. This condition severely damages chewing efficiency, nutrition intake, aesthetics and mental health and has an adverse effect on people's lives. Furthermore, periodontitis is closely related to multiple systemic diseases, such as atherosclerosis [3], diabetes mellitus (DM) [4], rheumatoid arthritis (RA) [5] and osteoporosis [6], and some patients with these conditions were reported to present more severe bone loss.

Alveolar bone is a dynamic and vibrant tissue. Normally, alveolar bone is subjected to mechanical stimulation and undergoes a continuous cycle of remodeling that is mainly based on the coordinated activities of two pivotal cell types: cells of the osteoblast lineage, including osteoblasts, osteocytes, and bone-lining cells, and bone-resorbing cells, such as osteoclasts. When osteoclasts are activated, they attach to the bone surface and produce protons (H^+^) and proteases to degrade the bone mineral matrix; thereafter, osteoblasts synthesize osteoid matrix and regulate the mineralization process. Under the regulation of various hormones and cytokines, bone resorption and bone formation are connected and balanced so that new bone can replace old bone, stabilizing the volume of alveolar bone [7]. However, in periodontitis, this balance is disrupted. Periodontal pathogen virulence overwhelms the oral mucosal defensive barrier and forces the epithelium to retreat apically on the root surface, resulting in the formation of a periodontal pocket. Resident cells, such as fibroblasts, keratinocytes and dendritic cells, within the tissue release inflammatory cytokines, which promote multiple inflammatory cells, such as neutrophils, macrophages, and T/B cells [8, 9], to migrate to the site of inflammation and gradually infiltrate deeper into the periodontal connective tissue, including alveolar bone. These inflammatory cells produce antimicrobial agents, reactive oxygen species, and enzymes to eliminate pathogens but also disrupt the normal activity of alveolar bone remodeling [10, 11]. Osteoclasts are induced and activated, while osteoblasts are inhibited, which disrupts the balance between bone removal and regeneration, leading to a reduction in bone volume [12].

Studies on the mechanism of bone loss in periodontitis have emphasized the role of osteoclasts and osteoblasts. However, some questions remain, such as what initiates bone remodeling in periodontitis, what directs osteoclast and osteoblast formation and localization, and how periodontitis interacts with systemic diseases. With the emergence of additional attention and studies on osteocytes, we have realized that there are some omissions and inaccuracies in our previous understanding. Abnormal osteocytes can lead to bone loss (Table 1). For example, targeted osteocyte ablation led to severe bone resorption and a fragile and sparse bone phenotype [13]. Adult-onset deletion of β-catenin from osteocytes can impact loading-induced bone adaptive reconstruction, contributing to decreased bone mass and density in mice [14]. Selective deletion of *Tnfsf11* (the gene encoding RANKL) in osteocytes resulted in a severe osteopetrotic phenotype [15]. Knock out of colony-stimulating factor (CSF)-1 in osteocytes increased osteocyte apoptosis due to the overproduction of intracellular reactive oxygen species and ultimately compromised bone formation and resorption, leading to increased cancellous bone [16]. Osteocyte-specific knockout of *Wnt1* caused low bone mass in mice, similar to that observed in osteogenesis imperfecta, because of a subsequent reduction in mammalian target of rapamycin complex 1 (mTORC1)-dependent osteoblast function [17]. These data suggested that the role of osteocytes in bone loss may extend beyond our current understanding. We should pay more attention to the functions of osteocytes, and this may provide new insights into the pathological processes underlying bone destruction in periodontitis.

**Table 1:** Specific gene deletion evidences on the role of osteocytes in bone resorption

Gene	Gene-editing method	Signaling pathway	Bone phenotype(s)	References
*β-catenin*	Deletion	Wnt/β-catenin signaling	Bone resorption↑, bone mass and density↓	[14]
*Tnfsf11*	Deletion	RANKL/RANK/OPG system	Bone resorption↓	[15]
*Csf-1*	Deletion	CSF‐1/Nox4/oxidative stress	Bone formation↓, bone resorption↓	[16]
*Wnt1*	Deletion	Wnt1-mTOR signaling	Bone formation↓	[17]
*Pgc-1*	Deletion	AMPK/PGC-1 pathway	Bone volume↓	[24]

## Osteocytes and their function

Osteocytes are one cell type that results from the terminal differentiation of osteoblasts and are the most numerous and long-lived cells in the bone. Osteocytes always occupy the lacunar space that is full of bone fluid, and osteocytes are characterized by an additional 50–60 cellular processes radiating from the cell body and extending through confined passages of canaliculi, all buried in the bone mineral matrix, which is also called the lacuno-canalicular system (LCS) [18]. Osteocytes interconnect cells, including each other and those over the bone surface, through dendritic processes coupled with gap junctions, which form the cellular functional network within the bone [19]. Osteocytes are versatile. Osteocytes act as orchestrators of bone remodeling in response to mechanical loading [20], changes in hormone levels [21], or other stimuli. A high prevalence of apoptosis in osteocytes induced by bone uploading was found to increase the expression of the osteocytic factor receptor activator of NF-κB ligand (RANKL), which exacerbated bone resorption in response to skeletal unloading [22]. Downregulation of sclerostin in osteocytes in response to parathyroid hormone (PTH) promotes osteoblastic activity and bone formation [23]. The AMP-activated protein kinase (AMPK)/proliferator-activated receptor co-activator-1 (PGC-1) pathway mediates low glucose-induced osteocytic gene expression, and conditional deletion of PGC-1 in osteocytes reduced the expression of osteocyte genes, such as dentin matrix protein (*Dmp1*), fibroblast growth factor 23 (*Fgf23*) and *Sost*, and consequently resulted in osteopenic phenomena [24].

**Fig. 1:**
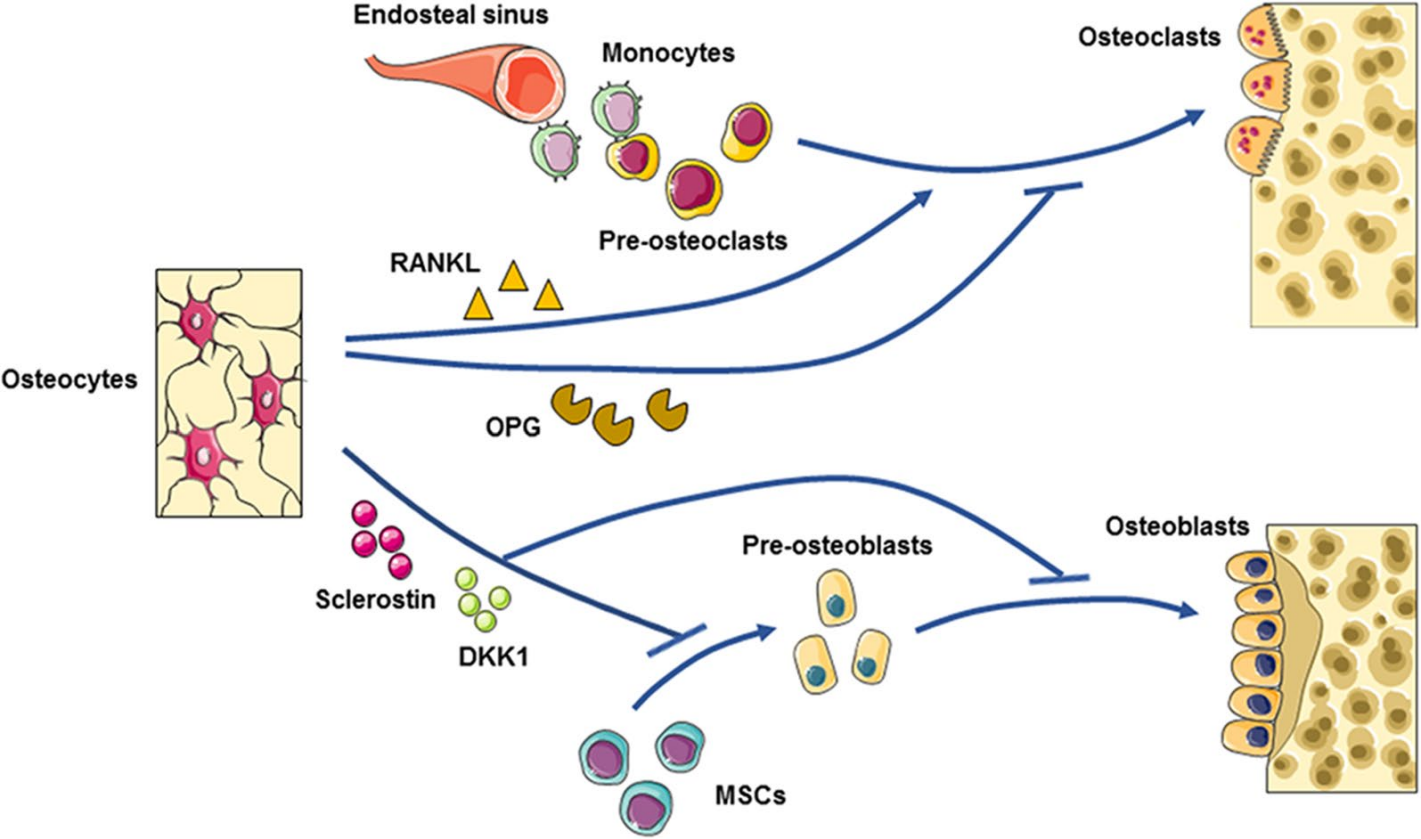
Osteocytes initiate bone remodeling through the regulation of osteoclasts and osteoblasts. (Top) Osteocytes express RANKL to promote, and OPG to inhibit, osteoclast generation and bone resorption. (Bottom) Osteocytes also secrete sclerostin and DKK1 to inhibit osteoblast formation and activity

Evidence has shown crosstalk among osteocytes, osteoclasts and osteoblasts and indicates that this crosstalk is required for physiological osseous turnover and homeostasis maintenance. On the one hand, osteocytes can affect osteoclasts. Osteocytes are the primary sources of RANKL, which acts upon receptor activator of NF-κB (RANK) and controls the differentiation of osteoclasts. RANKL expression is ten times higher in osteocytes than in osteoblasts and much higher in osteocytes than in other mesenchymal cells [15, 25]. Osteocytes can also predominantly produce osteoprotegerin (OPG), which prevents RANKL from activating osteoclastogenesis. Animate osteocytes can positively direct osteoclast formation and bone resorption by upregulating RANKL and downregulating OPG or, inversely, promote the opposite conditions to reduce bone resorption [26]. Additionally, dying osteocytes can control bone resorption. Osteocytes often undergo apoptosis in the context of bone microdamage, which is a principal trigger of site-specific resorption. For example, in osteocyte-specific connexin 43-knockout mice, increased osteocyte apoptosis and a lack of osteogenic and osteoclastic regulators, such as OPG and sclerostin, were observed, and these conditions increased endocortical resorption and periosteal bone formation [27]. On the other hand, osteocytes can affect osteoblasts in a stimulatory or inhibitory manner. Sclerostin and Dickkopf-related protein 1 (DKK1) are among the most potent signals originating from osteocytes. These molecules act as strong antagonists of Wnt/β-catenin signaling, which is an important pathway for promoting osteoblastogenesis and matrix formation [28, 29] (Fig. 1). Below, the recently elucidated role of osteocytes in the pathogenesis of periodontitis is discussed, highlighting RANKL and sclerostin secretion, osteocyte senescence, osteocyte apoptosis, and their potential effect on the association of periodontitis with systemic disease.

## The altered secretory function of osteocytes in periodontitis

### Osteocytes upregulate the expression of RANKL in periodontitis

**Fig. 2:**
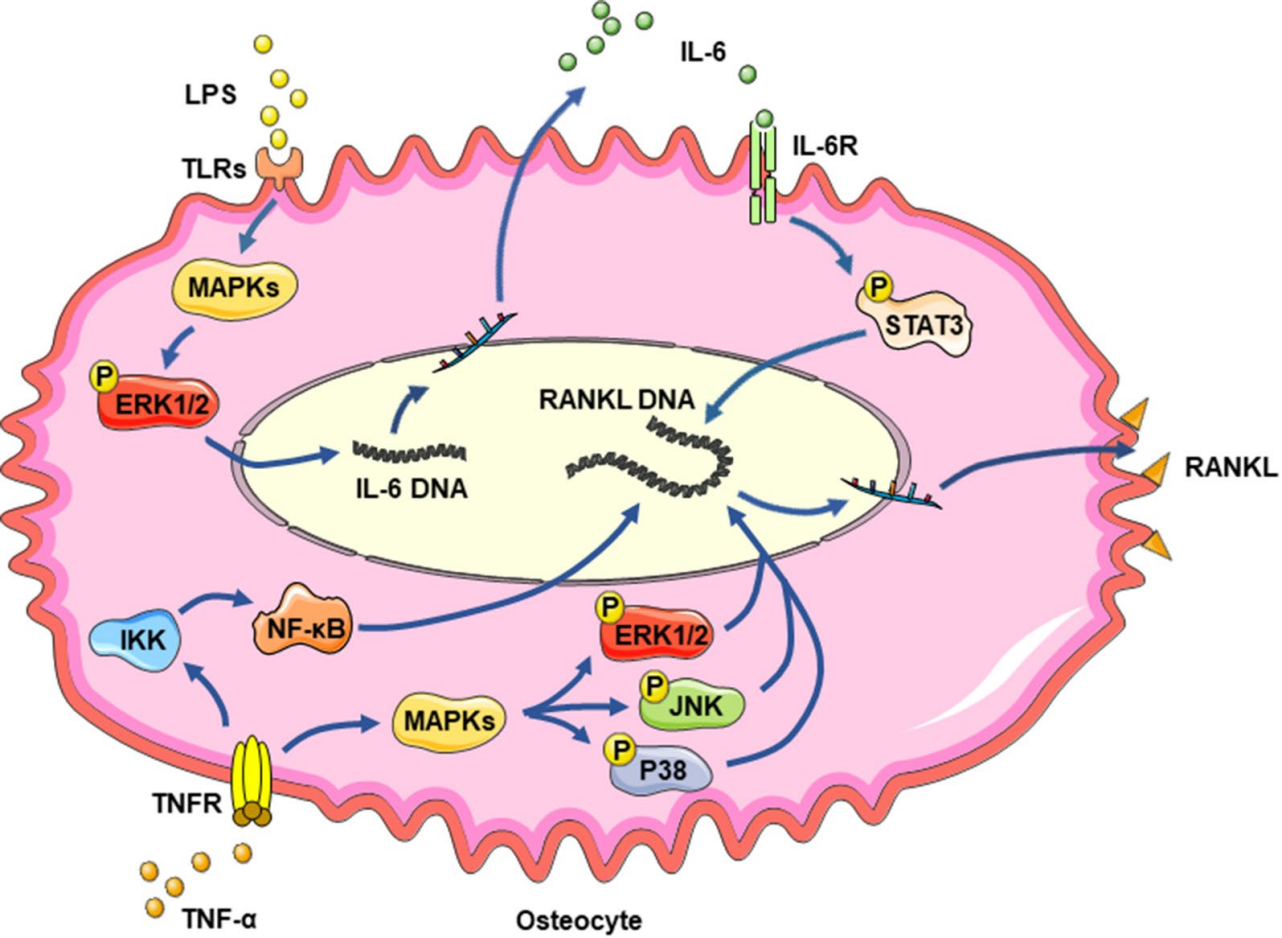
RANKL expression in osteocytes is upregulated under LPS and TNF-α stimulation. LPS binds to TLRs and activates the MAPK/ERK1/2 pathway, which promotes IL-6 production in osteocytes. IL-6 subsequently enhances RANKL expression by facilitating STAT signal transduction. TNF-α can promote RANKL generation through the NF-κB pathway and the ERK1/2, JNK and P38 MAPK pathways

Periodontitis tissue is flooded with many pathological factors, including biologically active substances within bacterial plaques and inflammatory mediators released by immune cells. Lipopolysaccharide (LPS) from gram-negative bacteria, which is recognized by Toll-like receptors (TLRs), such as TLR2, on the osteocyte surface irritates the downstream mitogen-activated protein kinase (MAPK)/extracellular signal-regulated kinase (ERK)1/2 signaling pathway and transcription factors, leading to upregulation of interleukin (IL)-6 expression [30]. IL-6 triggers gp130-mediated Janus kinase (JAK) activation, which then phosphorylates signal transducer and activator of transcription (STAT) [31]. Activated STAT translocates into the nucleus and ultimately increases RANKL expression in osteocytes [31, 32]. Inflammatory molecules from the host immune response, such as tumor necrosis factor-α (TNF-α) and IL-1β, are also conducive to increasing RANKL expression in osteocytes [33]. By binding to the TNF receptor on the osteocyte surface, TNF-α activates the ERK1/2, P38 and Jun kinase (JNK) MAPK signaling pathways and/or the transcription factor nuclear factor-kappa B (NF-κB), which enhances RANKL expression in osteocytes and consequently promotes alveolar bone resorption [34, 35] (Fig. 2). Many years ago, researchers discovered significantly enhanced RANKL content, along with downregulated OPG levels, in alveolar bone in periodontitis [36]. As the RANKL/OPG ratio increases, the quantity of osteoclasts increases accordingly, and the bone resorption area expands [37]. For a long time, we thought that T/B cells were the primary sources of RANKL in periodontitis [38, 39]. However, recent evidence has proven that osteocytes produce a large proportion of RANKL during bone remodeling in periodontitis [15, 40]. Induction of periodontitis stimulated a seven-fold increase in RANKL expression in murine osteocytes, consistent with the increased osteoclast number and bone resorption [35]. Through the utilization of transgenic model mice, scholars have observed the unique activity of osteocyte-produced RANKL in periodontitis bone resorption [8].

RANKL is a TNF superfamily protein. RANKL serves as a key regulator of osteoclastogenesis and functions as a membrane-bound or soluble form. The combination of RANKL and RANK on the cytomembrane of osteoclast precursors and osteoclasts is required for osteoclast fusion, differentiation, activation and subsistence [37], and the membrane-binding form of RANKL serves in a dominant way [41]. It has long been recognized that the content of RANKL is strongly related to the severity of bone resorption. OPG, which is mainly expressed by cells of the osteoblast lineage, is a soluble decoy receptor of RANKL. OPG competes with RANK to inhibit RANKL/RANK binding to restrain the activation of osteoclasts [26, 37].

Membrane-bound RANKL is essential for bone resorption. Because osteocytes are embedded deep in the hard bone matrix, how RANKL on the osteocyte surface interacts with preosteoclasts located on the bone surface is unknown the answer to this question involves the subcellular transport of RANKL. The use of a combination of a collagen-embedded 3D culture system and a porous membrane separating osteocytes from bone marrow macrophages (BMMs) of C57BL/6 J mice facilitated the investigation of the regulatory mechanism of RANKL transport in osteocytes under the premise of maintaining the physiological morphology of bone cells [41]. It was concluded that osteocytes directly interact with BMMs via the extremities of their dendritic processes and provide the latter with RANKL, which was promoted upon stimulation with RANK-conjugated beads. Furthermore, RANKL is mainly concentrated in osteocyte lysosomes, and OPG is required for regulation of RANKL trafficking [41].

At present, drugs designed to inhibit RANKL in order to inhibit osteoclast activity and prevent bone resorption have been developed. Denosumab, an anti-RANKL monoclonal antibody, is currently being used to treat patients with osteoporosis at high risk of hip fracture and the progression and metastasis of cancer and giant cell tumors within bone [42]. However, these drugs are still in preclinical trials for treatment of periodontitis. In some studies, the application of a monoclonal antibody specific for RANKL was demonstrated to be effective in ameliorating alveolar bone destruction in experimental periodontitis and LPS-induced calvarial bone damage in a mouse model; the coverage of osteoclasts in calvaria was greatly diminished, and the amount of resorption pits caused by osteoclasts was significantly reduced compared to that in the control group [43]. The same therapeutic outcome was shown in a mouse model of periodontitis treated with a human recombinant OPG fusion protein [44]. However, traditional anti-resorption drugs can cause serious complications, such as osteonecrosis of the jaw (ONJ) and atypical femoral fractures [45]. Due to suppressed osteoclast function, bone turnover is reduced, resulting in diminished mineral density and serum calcium [46, 47]. Jaw bones are susceptible to adverse drug reactions because they are subjected to constant mechanical stress, undergo frequent remodeling and have relatively high vascularity, which may partly explain the specific location of ONJ [48]. These side effects limit the use of anti-resorption medication in the treatment of periodontal disease. Therefore, more research is needed to improve the application of anti-RANKL therapy in periodontitis to lower alveolar bone loss.

### Osteocytes generate more sclerostin and DKK1 in periodontitis

**Fig. 3:**
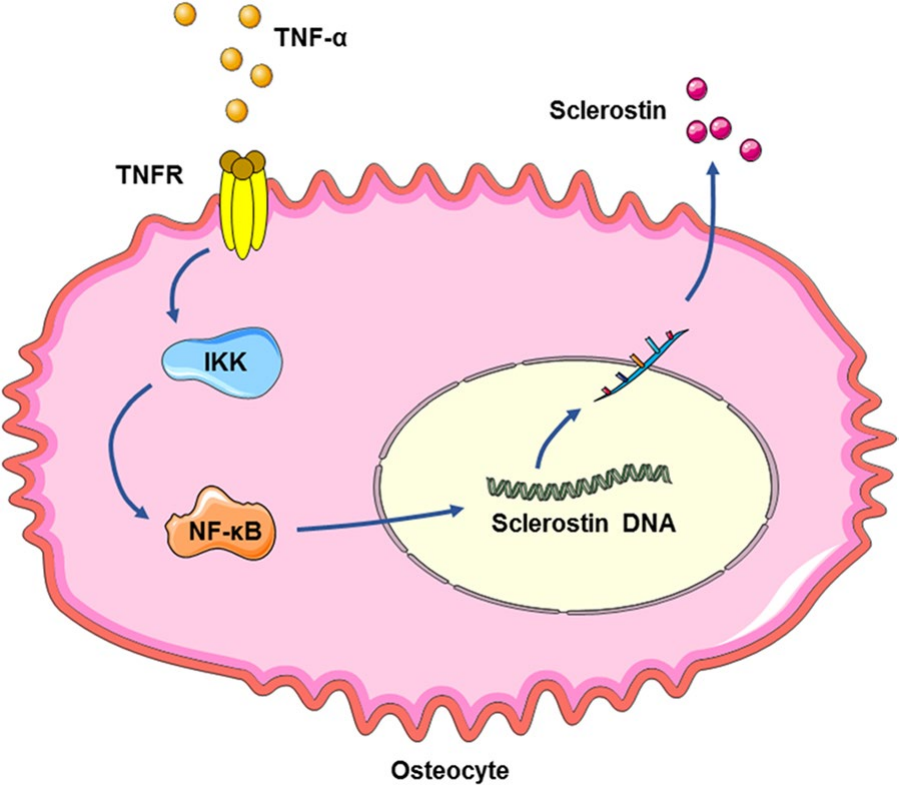
TNF-α mediates sclerostin expression in osteocytes via the NF-κB pathway

The mechanism of bone loss in periodontitis involves not only the enhancement of osteoclast activity but also the weakening of osteoblast activity, which exacerbates the progressive destruction of alveolar bone. Proinflammatory cytokines, such as TNF-α and IL-1β, can induce sclerostin expression in osteocytes. TNF-α can enhance sclerostin expression through an NF-κB-dependent mechanism, during which NF-κB directly binds to the *SOST* promoter region and induces an increase in sclerostin expression [35, 49] (Fig. 3). DKK1 can enhance the sclerostin expression induced by TNF-α in osteocytes to inhibit osteoblast activity and impair bone formation [50]. Evidence related to the effects of periodontitis bacteria and their toxic substances on sclerostin production in osteocytes is insufficient. LPS may indirectly promote sclerostin production by stimulating osteocytes to produce TNF-α, IL-6 and other pro-inflammatory factors, but no evidence has been found to show its direct effect [51].

The sclerostin content in gingival crevicular fluid from chronic periodontitis patients markedly exceeds that of healthy subjects [52], and the protein levels of sclerostin and DKK1 in gingival biopsy tissue and serum are relatively increased [53, 54]. In animal experiments, an increase in sclerostin-positive osteocytes within alveolar bone was linked to a decrease in bone formation in a mouse model of periodontitis [55, 56]; in parallel, the administration of sclerostin- and DKK1-specific antibodies markedly improved alveolar bone volume and structure [57, 58].

Sclerostin, encoded by the *SOST* gene, is a kind of secreted glycoprotein primarily generated by osteocytes. In *Sost* knockout mice, bone thickness, bone density, and bone mechanical strength are enhanced [59], while transgenic overexpression of *Sost* led to osteopenia [60]. Sclerosteosis and van Buchem disease [61, 62], both caused by an absence of sclerostin, lead to excessive bone growth. DKK1 is another type of endogenous secretory protein mainly produced by osteocytes. Both sclerostin and DKK1 can negatively mediate osteoblastogenesis and osteoblastic activity by interrupting Wnt/β-catenin signaling, and they compete with WNT proteins for binding to the extracellular regions of low-density lipoprotein receptor-related protein-5/6 (LRP5/6) on osteoblasts [63, 64]. DKK1 also has a catabolic function and decreases the expression of OPG by inhibiting the Wnt/β-catenin signaling pathway, leading to an increased local ratio of RANKL/OPG in osteocytes, which increases osteoclastogenesis and osteoclast activity and promotes bone absorption [65–68].

Pharmaceutical therapy against sclerostin with a neutralizing monoclonal antibody (Scl-Ab) have been used for the clinical treatment of certain diseases, including osteoporosis and fracture. Romosozumab, an Scl-Ab agent, has completed phase III studies. Romosozumab was found to be able to reduce the threat of vertebral, hip, and other fractures in postmenopausal women suffering from osteoporosis [69, 70]. Many studies suggest that Scl-Ab has promising prospects in the treatment of periodontitis. A mouse model with ligature-induced experimental periodontitis exhibited decreased bone volume and tissue mineral density in the local alveolar area, while after three weeks of systematic application of Scl-Ab, the indexes showed a trend of recovery and were completely reversed after six weeks, with no significant differences from the healthy control mice [71]. In parallel, a combination of anti-sclerostin and anti-DKK1 antibodies markedly improved alveolar bone volume and structure [57, 58], while the effect of local injection of Scl-Ab on alveolar bone recovery was limited. Similar outcomes were obtained in other studies [71–73]. In a preclinical study, the application of Scl-Ab following osteoporotic doses in the treatment of periodontitis in ovariectomized rats did not lead to ONJ [74]. The authors considered that the local inflammatory microenvironment in periodontitis may overcome the systemic osteoclast inhibition caused by sclerostin inhibition. Moreover, it has been reported that ONJ also presents in postmenopausal women with osteoporosis during romosozumab therapy. More and longer studies are required.

## Potential effect of premature osteocyte senescence on bone remodeling in periodontitis

Osteocyte senescence is common in older individuals and is directly related to age-related bone loss or osteoporosis [75]. A small fraction of senescent cells (for instance, approximately 10–15% in aged primates) are conducive to tissue malfunction [76, 77]. However, cellular senescence can also occur in young individuals and is called premature senescence. Cellular senescence is a stress reaction to physical, chemical or biological stimulation, and it terminates the proliferation of dysfunctional cells and puts them in a state of irreversible growth stagnation [78, 79]. This involves a series of transformations in cellular morphology and functions, such as gene expression and metabolism, as well as the development of a proinflammatory secretome or senescence-associated secretory phenotype (SASP) [80]. The accumulation of senescent osteocytes in young alveolar bone with respect to the ramus has been observed by measuring senescence-associated distension of satellites, a marker of cellular senescence, accompanied by upregulation of SASP expression [81]. Given that alveolar bone is in close contact with periodontal pathogens and their products for a long period, researchers [81] assumed that this may account for the advanced senescence of osteocytes. After in vitro and ex vivo experiments, researchers observed that LPS exposure can cause DNA damage and premature senescence in osteocytes and the resulting release of SASP through activation of P53 [81]. It is unclear whether premature LPS-induced osteocyte senescence has an influence similar to that of age-related osteocyte aging on bone remodeling and how its effects are mediated or if it acts in a specific way.

**Fig. 4:**
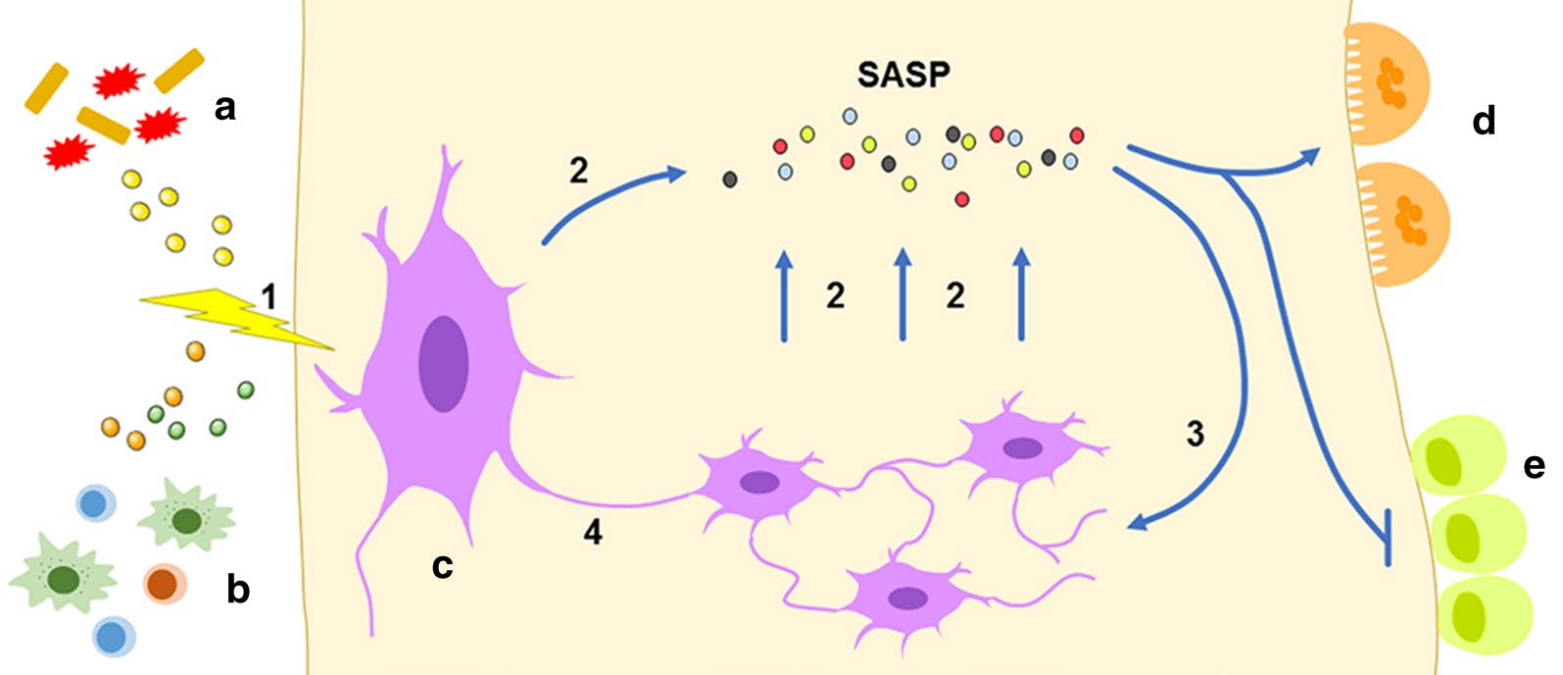
Premature osteocyte senescence and its downstream effects in periodontitis. In periodontitis, stimulation by bacteria (**a**) and inflammatory cells (**b**) drives osteocytes to undergo premature senescence (1). Senescent osteocytes (**c**) express SASP (2), which reinforces osteoclast (**d**)-mediated bone resorption but inhibits osteoblast (**e**)-mediated bone formation. Senescent osteocytes can induce senescence in adjacent cells through SASP (3) and/or cell–cell contact (4)

Osteocytes are the initiators of bone remodeling. Owing to the consistent pathological factors and inflammatory stimulation in periodontal tissue, osteocytes tend to become senescent and subsequently dysfunctional. This process is called inflamm-aging and may jeopardize bone remodeling and bone homeostasis [82, 83]. SASP, which is comprised of a variety of secretory proteins, including inflammatory cytokines, chemokines, extracellular matrix remodeling factors, and growth factors, is an important characteristic of senescent cells and has benefits, such as tumor suppression [84], but typically, it promotes chronic inflammation and/or tumorigenesis [85, 86]. Indeed, many SASP elements, such as IL-1β, IL-6, TNF-α, and matrix metallopeptidase-13, were shown to inhibit osseous formation and promote osseous resorption [87, 88]. Thus, senescent cells may establish a secondary source of inflammatory factors and proteolytic factors that exacerbate alveolar bone destruction. Neutralization of these SASP components with antibodies can attenuate the pro-osteoclastogenic effects of senescent cells and restrict the generation of osteoclasts [76]. In addition, SASP is released by osteocytes in an autocrine and paracrine manner and induces secondary senescence in adjacent cells exposed to it, thereby exacerbating and amplifying the senescence and inflammatory states [89]. In the context of age-related changes in bone, senescent osteocytes exhibit dramatic degeneration of cellular processes and diminished osteocyte density, resulting in vacancies in the overall cellular connection network, which could further cause defective mechanotransduction, impaired nutrient acquisition and affected intercellular signal transduction, resulting in significant bone loss [90, 91]. In addition to expressing SASP, senescent osteocytes are also capable of inducing DNA damage and propagating senescence to bystander cells through gap junction-dependent intercellular connections; this propagated senescence is termed senescence-induced senescence, and it creates a toxic inflammatory microenvironment to accelerate and spread its effects [89, 92] (Fig. 4). Overall, the osteocyte senescence discovered in periodontitis and its possible repercussions may contribute to alveolar bone loss. These findings may partially explain why age acts as a risk factor for periodontitis, and treatment targeting senescent osteocytes may provide a new method to delay the progression of bone destruction in periodontitis.

## Osteocyte apoptosis in periodontitis

In periodontitis, the apoptosis of inflammatory cells and periodontium cells, including osteocytes, is increased, and the apoptosis of these cells exerts a significant effect on the progression of chronic inflammation and tissue damage [93, 94]. Apoptosis is a type of programmed cell death mediated by gene expression. Apoptotic cells undergo degradation of chromatin and organelles, but the integrity of cytoplasmic membranes is maintained, and these membranes develop into apoptotic bodies and await phagocytosis and clearance by macrophages. In the last century, it has been observed that osteocytes can die very early in certain physiological or pathological conditions [95]. The death of osteocytes can modify the targeting of osteoclastogenesis followed by increased bone resorption in defined areas [13, 40, 96].

**Fig. 5:**
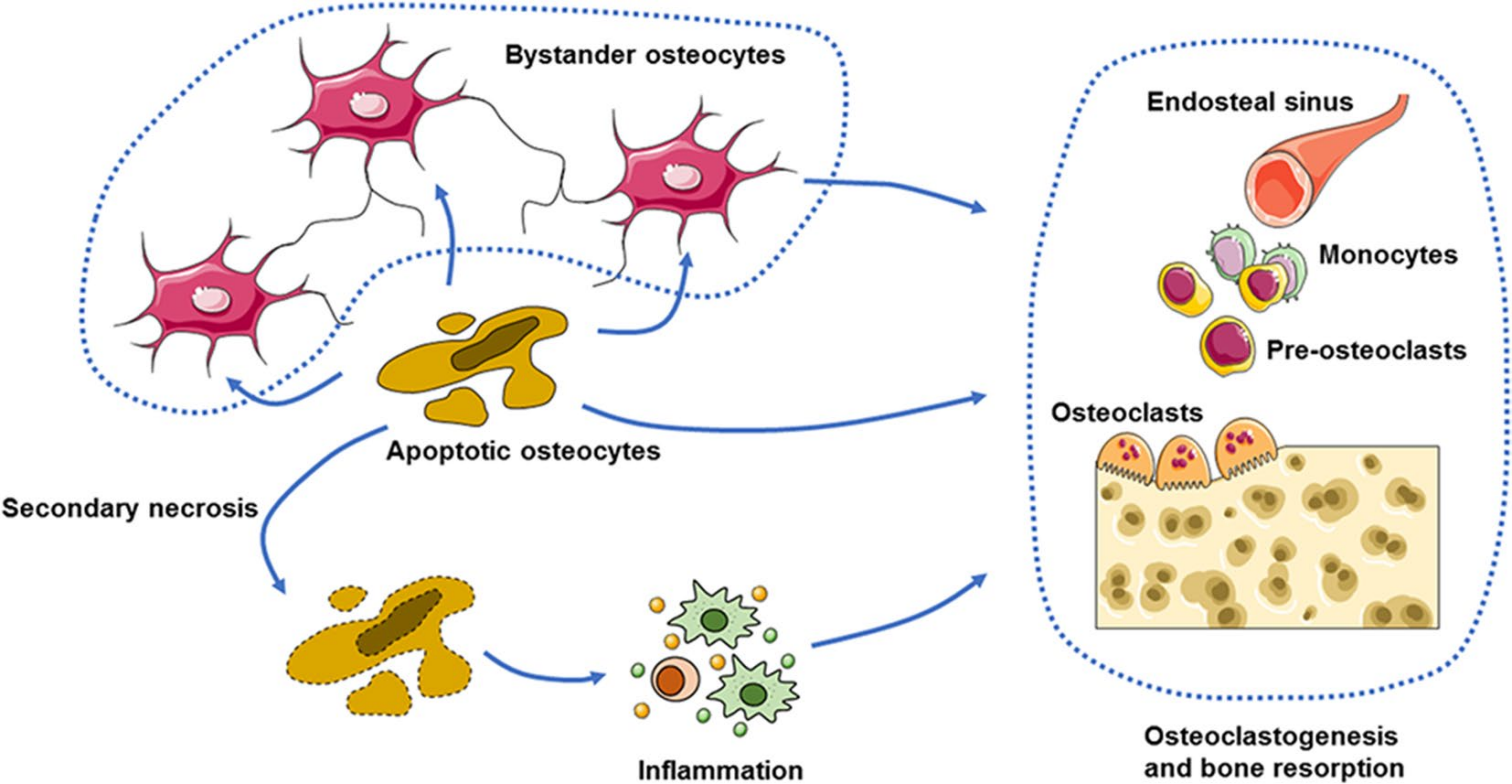
Apoptosis of osteocytes directly or indirectly promotes osteoclastogenesis and related bone resorption. Apoptotic osteocytes can release, or induce surrounding osteocytes to express, RANKL to modulate preosteoclast adherence and differentiation into osteoclasts. Secondary osteocyte necrosis can induce local inflammation and promote osteoclast formation and activation

The impact of osteocyte apoptosis can be propagated through the secretion of multiple cytokines. Osteocyte populations comprising apoptotic osteocytes produce more IL-6 and soluble IL-6 receptor (sIL-6R). IL-6 can bind to sIL-6R and then form a high-affinity heterotrimer with the ubiquitously expressed transmembrane gp130, which is termed IL-6 trans signaling and widens the spectrum of affected cells to include those that do not have endogenous IL-6R [97]. When IL-6 and SIL-6R interact, the cytoplasmic domain of gp130 binds to JAK and triggers subsequent signaling cascades, such as the JAK/STAT pathway [31], which then activates endothelial cells to increase the expression of intercellular adhesion molecule-1 (ICAM-1), allowing osteoclast precursor cells to adhere to the vascular endothelium [98]; this is an important premise to approach the site of reconstruction. However, IL-6 can also mediate signal transduction through another alternative pathway known as classic signaling, in which a high-affinity heterotrimer forms with the membrane-bound IL-6 receptor (IL-6R) and gp130 and incorporates JAK to activate downstream signaling [31]. According to some studies, endothelial cells express little membrane-bound IL-6R, and trans-signaling may play a dominant role in this process [99]. Apoptotic osteocytes can also release adenosine triphosphate (ATP) through activated pannexin 1 channels, and ATP acts on osteocytes, osteoblasts, osteoclasts and macrophages via ATP receptor-gated (P2) channels and consequently induces enhanced production of RANKL in bystander osteocytes and osteoblasts, aggregation of macrophages, and increased osteoclastogenesis [40, 100, 101] (Fig. 5).

In addition to cytokine secretion, structural components produced by apoptotic osteocytes can also affect bone remodeling. Apoptotic bodies of dying osteocytes can promote osteoclast progenitor cells to differentiate into osteoclasts. Apoptotic osteocytes can secrete RANKL and directly modulate osteoclast formation and bone remodeling through RANKL secretion [102, 103], but in contrast, some evidence indicates that this process is not entirely dependent on RANKL because the osteoclastogenesis induced by osteocyte apoptotic bodies is not decreased in the presence of OPG concentrations ranging from 50 ng/mL to higher levels (> 400 ng/mL). Interestingly, TNF-α produced by mononuclear osteoclast precursor cells that recognize specific markers exposed on the membranes of apoptotic osteocytes appears to be responsible for enhanced osteoclast formation [104, 105]. In addition, if apoptotic osteocytes are not cleared by macrophages in a timely manner, the cytoplasmic membrane ruptures, and this is termed secondary necrosis. This process subsequently provokes the secretion of multiple immunostimulatory cytokines and the aggregation and energization of immune cells, such as macrophages, monocytes, dendritic cells and neutrophils, which potentiates the generation of proinflammatory molecules and stimulates the secretion of RANKL in neighboring cells [101, 106].

At present, the reason for osteocyte apoptosis in periodontitis and its relationship to alveolar bone damage are still not clear. However, few studies have reported that bacterial stimulation and inflammatory factors can promote osteocyte apoptosis. Gingipains, toxic proteolytic enzymes secreted by *Porphyromonas gingivalis*, can induce transmembrane integrin β1 degradation and inhibit Rho family GTPases, which account for the depolymerization of the cytoskeletal protein F-actin and induce the deficiency of adherence between cells and the extracellular matrix, resulting in osteocyte apoptosis [107]. The inflammatory environment in periodontal tissue is also a main cause of cell apoptosis, and the inflammatory environment caused by the death of cells can also promote osteocyte apoptosis. In periodontitis, IL-1, IL-6 and TNF-α are common inflammatory cytokines confirmed to modulate osteocyte apoptosis. In an exploration of the mechanism of homocysteine (Hcy)-induced bone fragility, Hcy was found to increase IL-1β and IL-6 expression by increasing NADPH oxidase (Nox) 1 and Nox2 expression, thus contributing to apoptosis of osteocyte-like cells [108]. In multiple myeloma, TNF-α generated by tumor cells is augmented and amplifies apoptosis of osteocytes, while neutralization of TNF-α failed to completely reverse this effect [109, 110]. Furthermore, given that osteocytes are the terminal differentiation form of osteoblasts, the mechanism of osteoblast apoptosis may also provide some insight into that of osteocytes. It has been demonstrated that IL-1α promotes osteoblast apoptosis and suppresses osteoblast differentiation through the activation of the JNK and p38 MAPK pathways, resulting in the exacerbation of inflammation-related bone destruction [111–113]. TNF-related apoptosis-inducing ligand (TRAIL) can also mediate osteoblast apoptosis in periodontitis owing to an imbalance between the death and decoy receptors of TRAIL, which activates caspase-8 and caspase-3 and consequently causes DNA degeneration [114]. These mechanisms may also provide another cause of osteocyte apoptosis. In addition to apoptosis, the functions of other death mechanisms, such as autophagy, necroptosis and nonprogrammed death, in periodontitis require further study. Elucidating the role of osteocyte death in periodontitis will bring positive significance to our comprehensive understanding of the occurrence and development of periodontitis and help to improve future clinical prevention, diagnosis and treatment of periodontitis.

## Osteocytes may participate in the communication between periodontitis and systemic diseases

Periodontitis is not a simple disease that merely sweeps across the oral cavity, but rather, it has numerous correlations with general health. Systemic diseases, including DM, cardiovascular disease (CVD), RA and osteoporosis, share a bidirectional relationship with periodontitis [4, 115]. Disorders of the internal environment caused by systemic diseases, including inflammatory state, oxidative stress, and hormone secretion dysfunction, can exacerbate the local inflammatory response and tissue destruction in periodontitis; moreover, the continuous inflammatory condition and the entrance of bacteria or their products into blood circulation may be a mechanism by which periodontitis provokes systemic pathosis [116–118]. Bone is a tissue with high vascularity, and the osteocyte network has abundant connections with blood vessels, which provides a channel for interaction between the overall internal environment and the skeletal microenvironment. The osteocyte network participates in intercommunication between bone and distant organs, such as the kidneys, muscles and other organs, and is closely related to the regulation of disease status, such as cancer metastasis [119], diabetes-related bone loss [120], and chronic kidney disease (CKD) [121] (Table 2). For example, hyperglycemia and advanced glycation end-products (AGEs) that emerge in the chronic hyperglycemia state of DM can increase the production of sclerostin in osteocytes [51, 122]. In CKD, the apoptosis of osteocytes increases, and the expression of DMP1 decreases, followed by the upregulation of FGF23 in osteocytes, which contributes to phosphate wasting and osteomalacia [123]. The overproduction of sclerostin in osteocytes caused by a decrease in PTH levels is considered to be one of the reasons for the reduction in bone formation and mineralization failure after kidney transplantation [124]. In CKD model mice, conditional knockout of the PTH/PTH-related protein type 1 receptor (PPR) can exacerbate osteogenic deficiency at the endocortical surface but reverse bone loss on the periosteal surface [125]. In turn, osteocytes can influence the development of systemic diseases. It has been shown that conditioned medium from osteoclasts and vascular endothelial cells treated with conditioned medium from loaded osteocytes can inhibit the metastasis and promote the apoptosis of breast cancer cells [126]. In most RA and osteoarthritis (OA) patients, the expression of cyclooxygenase-2 (COX-2) in osteocytes is high [127]. Induced overexpression of osteocyte COX-2 caused spontaneous OA and transgenic RA, and exclusive knockout of *Cox-2* in osteocytes attenuated joint cartilage degeneration [127]. The loss or mutation of DMP1 or phosphate-regulating gene with homologies to endopeptidases on the X chromosome (Phex), which are both highly expressed by osteocytes, can lead to upregulation of FGF23 in osteocytes and subsequent hypophosphatemia [128, 129]. Notably, an evolving concept, termed “osteoimmunology”, may play a role in this relationship. The skeletal and immune systems share many regulatory factors, which form the basis of the interaction between bone and the immune system. Some systemic diseases, such as DM and RA, can cause chronic systemic inflammation, contributing to the activation of immune cells and elevation of inflammatory substances [130, 131], which can exacerbate the disruption of bone homeostasis in periodontitis. For example, type 1 diabetes can elevate the proportion of T helper type 17 cells in the immune system and lead to an increase in the secretion of IL-17 [132, 133], which is a known proinflammatory and osteoclastogenic factor that promotes RANKL production in osteoblasts [134, 135]. However, the specific role of osteocytes in the immune system is still not clear. Based on the above, osteocytes may provide a new approach to describe the interplay of periodontitis and systemic disease, and we recommend future research focusing on the junctional role of the osteocyte network.

**Table 2:** The role of osteocytes in periodontitis and other systemic diseases

Influence factor	Alteration in osteocytes	Signaling pathway	Phenotype(s)	Disease status	References
PPR knockout	–	–	Endocortical bone formation↓, periosteal bone formation ↑	CKD	[125]
DMP1 or Phex loss/mutation	FGF23↑	–	Phosphate wasting↑, bone mineralization↓	Hypophosphatemic rickets	[128, 129]
Apoptosis of osteocytes	IL-6↑	IL-6/JAK/STAT	I-CAM1 in endothelial cells↑, osteoclast adherence↑	Periodontitis	[98]
	ATP↑	–	RANKL↑ (in bystander cells)	Periodontitis	[40, 100, 101]
	Apoptotic bodies	–	RANKL↑(in bystander cells), TNF-α↑ (in osteoclast precursor cells) Osteoclast differentiation↑	Periodontitis	[102, 103]
LPS	Il-6↑, RANKL↑	MAPK/ERK1/2, IL-6/JAK/STAT	Osteoclast differentiation↑, bone resorption↑	Periodontitis	[32]
TNF-α	RANKL↑	MAPK/ERK1/2/p38/JNK, NF-κB signaling	Osteoclast differentiation↑, bone resorption↑	Periodontitis	[34, 35]
	Sclerostin↑	NF-κB signaling	Osteoblast activity↓, bone formation↓	Periodontitis	[35, 49]
Hyperglycemia and AGEs	Sclerostin↑	–	Osteoblast activity↓, bone formation↓	DM-related periodontitis	[51, 122]
PTH↓	Sclerostin↑	–	Bone formation↓, bone mineralization↓	Kidney transplantation complication	[124]
Loading	–	–	Metastasis↓, apoptosis↑ (in breast cancer cells)	Breast cancer	[126]
–	COX-2↑	–	Joint cartilage degeneration↑	OA, RA	[127]
–	DMP-1↑, FGF23↓	–	Phosphate wasting↑, osteomalacia	CKD	[123]

## The role of other cells with osteoblast-like characteristics in bone loss in periodontitis

In addition to osteocytes, other cells with osteoblastic properties may also be involved in periodontitis-related bone destruction. Periodontal ligament cells (PDLCs), capable of expressing alkaline phosphatase and bone-associated proteins, are osteoblast-like cells [136]. It has been reported that PDLCs can enhance osteoclast formation and accelerate bone resorption in response to certain stimuli through the enhanced activation of the TLR4 pathway and higher expression of inflammatory cytokines, including RANKL, TNF-α, macrophage-colony stimulating factor, IL-6, IL-1β, and CXC motif chemokine 2 [137–140]. The ability of PDLCs to promote inflammation and osteoclast formation can be inhibited when NF-κB signaling is inhibited by the induction of the exclusive expression of a dominant negative mutant of Iκ kinase [140]. This characteristic of PDLCs can also be enforced by the inhibition of orthodontic force-induced tooth movement and osteoclastogenesis in transgenic mice in which RANKL was deleted in PDLCs and bone lining cells [141]. In addition to PDLCs, gingival fibroblasts (GFs) can also be stimulated to differentiate into the osteogenic lineage, although their osteogenic capacity is weaker than that of PDLCs [142]. GFs can express osteopontin and osteocalcin, leading to mineralization under dexamethasone treatment [143]. To some extent, GFs also have the potential to inhibit osteoclastogenesis compared to PDLCs [144]. Stimulating GFs with TLR2 and TLR4 agonists does not affect their osteogenic capacity but significantly inhibits their ability to promote osteoclast formation [145].

Furthermore, periodontal ligament stem cells (PDLSCs) and gingival mesenchymal stem cells (GMSCs) also exhibit enhanced osteogenic differentiation potential and are widely studied in skeletal tissue engineering research [146]. PDLSCs can exhibit increased proliferation and mineral deposition in response to some treatments, such as kaempferol and curcumin [147, 148]. GMSCs can adhere to a titanium surface and deposit mineralized tissue, which resembles native bone [149]. In combination with biocompatible materials, these cells show promising application prospects in periodontal bone regeneration [150, 151].

These results suggest that other periodontal cells with osteoblast-like characteristics, such as osteoblasts, osteoclasts and osteocytes, may also play an important role in the destruction of alveolar bone. Elucidating their roles will help us better understand the pathological mechanism of periodontitis and provide new targets for treatment of periodontitis.

## Conclusions

In recent years, the regulatory effect of osteocytes in physiological and pathological processes has received increasing attention. Here, we summarize the latest research discoveries on the mechanism by which osteocytes promote alveolar bone loss in periodontitis. Osteocytes, with their unique structural and functional characteristics, are important for pathological alveolar bone remodeling in periodontitis and may facilitate the interaction between periodontitis and systemic diseases. Other cells with osteoblastic properties may also play a role in promoting periodontitis development. Hence, research on osteocytes and other cells could shed light on the mechanism underlying tissue destruction in periodontitis and provide a new target for the effective management and treatment of periodontitis as well as tissue regeneration therapy.

## Data Availability

Not applicable.

## Abbreviations

AGEs: Advanced glycation end-products
AMPK: AMP-activated protein kinase
ATP: Adenosine triphosphate
BMMs: Bone marrow macrophages
CKD: Chronic kidney disease
COX-2: Cyclooxygenase-2
CSF: Colony-stimulating factor
CVDs: Cardiovascular disease
DKK1: Dickkopf-related protein 1
DM: Diabetes mellitus
DMP1: Dentin matrix protein 1
ERK: Extracellular signal-regulated kinase
FGF23: Fibroblast growth factor 23
GFs: Gingival fibroblasts
GMSCs: Gingival mesenchymal stem cells
F-actin: Actin filaments
Hcy: Homocysteine
ICAM-1: Intercellular adhesion molecule-1
IL: Interleukin
IL-6R: IL-6 receptor
JAK: Janus kinase
JNK: Jun kinase
LCS: Lacuno-canalicular system
LPS: Lipopolysaccharide
LRP5/6: Low-density lipoprotein receptor-related protein-5/6
MAPK: Mitogen-activated protein kinase
NF-κB: Nuclear factor-kappa B
Nox: NADPH oxidase
OA: Osteoarthritis
ONJ: Osteonecrosis of the jaw
OPG: Osteoprotegerin
PDLCs: Periodontal ligament cells
PDLSCs: Periodontal ligament stem cells
Phex: Phosphate-regulating gene with homologies to endopeptidases on the X chromosome
PPR: PTH/PTH-related protein type 1 receptor
PTH: Parathyroid hormone
RA: Rheumatoid arthritis
RANK: Receptor activator of NF-κB
RANKL: Receptor activator of NF-κB ligand
SASP: Senescence-associated secretory phenotype
Scl-Ab: Sclerostin antibody
sIL-6R: Soluble IL-6 receptor
STAT: Signal transducer and activator of transcription
TLR: Toll-like receptor
TNF-α: Tumor necrosis factor-α
TRAIL: TNF-related apoptosis-inducing ligand
